# Stigmatizing and discriminatory attitudes toward people living with HIV/AIDS (PLWHA) among general adult population: the results from the 6^th^ Thai National Health Examination Survey (NHES VI)

**DOI:** 10.7189/jogh.13.04006

**Published:** 2023-01-14

**Authors:** Sineenart Chautrakarn, Parichat Ong-Artborirak, Warangkana Naksen, Aksara Thongprachum, Jukkrit Wungrath, Suwat Chariyalertsak, Scott Stonington, Surasak Taneepanichskul, Sawitri Assanangkornchai, Pattapong Kessomboon, Nareemarn Neelapaichit, Wichai Aekplakorn

**Affiliations:** 1 Faculty of Public Health, Chiang Mai University, Chiang Mai, Thailand; 2 Departments of Internal Medicine and Anthropology, University of Michigan, Michigan, USA; 3 College of Public Health Sciences Chulalongkorn University, Bangkok, Thailand; 4 Faculty of Medicine, Prince of Songkla University, Songkla, Thailand; 5 Faculty of Medicine, Khon Kaen University, Khon Kaen, Thailand; 6 Faculty of Medicine Ramathibodi Hospital, Mahidol University, Bangkok, Thailand; 7 National Health Examination Survey Office, Bangkok, Thailand

## Abstract

**Background:**

Thailand has an ongoing action plan to reduce human immunodeficiency virus (HIV) discrimination and stigma. We aimed to monitor the level of stigmatizing and discriminatory attitudes toward people living with HIV/AIDS (PLWHA) among the general adult population and to investigate its related factors.

**Methods:**

This study was based on data from the 6^th^ Thai National Health Examination Survey, a large-scale country-wide survey in 2019-2020. We used a multistage sampling technique and included 11 843 adults aged 20 to 59. We collected data through face-to-face interviews which included six items related to HIV stigma domains. We weighted all analyses to account for the probability of sampling the Thai population aged 20 to 59 years.

**Results:**

We found that anticipated stigma had the highest percentage of negative stigmatizing attitude responses (78.5%), followed by perceived stigma (66.6%), fear of HIV infection (54.4%), and social judgment (28.2%). Regarding the UNAIDS global indicator for discriminatory attitude, 48.6% of respondents had negative perceptions to questions about experienced stigma or discrimination. Multiple logistic regression showed that factors associated with discriminatory attitudes toward PLWHA were being aged 20-39 (adjusted odds ratio (aOR) = 1.32, 95% confidence interval (CI) = 1.18-1.47) or 50-59 (aOR = 1.23, 95% CI = 1.09-1.40) compared to being aged 40-49, being Muslim compared to Buddhist (aOR = 1.73, 95% CI = 1.46-2.06), being married compared to being single (aOR = 1.15, 95% CI = 1.04-1.28), holding certificate degree or higher compared to not studying or studying at a primary level (aOR = 0.81, 95% CI = 0.68-0.97), living in the Northeast (aOR = 1.27, 95% CI = 1.12-1.45) and Bangkok (aOR = 1.30, 95% CI = 1.12-1.51) compared to living in the North, having no HIV/AIDS infected relative or acquaintance compared to having an HIV/AIDS infected relative or acquaintance (aOR = 1.56, 95% CI = 1.41-1.73), and not obtaining an HIV test compared to obtaining it (aOR = 1.10, 95% CI = 1.02-1.19).

**Conclusions:**

We found that HIV stigmatizing and discriminatory attitudes toward PLWHA decreased, but remained concerning among Thai adult people. A public education and awareness campaign, as well as an intervention to reduce HIV-related stigma and discrimination in the country's health care facilities, must still be maintained.

Despite over three decades of progress, human immunodeficiency virus (HIV) remains a major global public health issue. In 2018, an estimated 37.9 million people were living with HIV, with 1.7 million new infections and 770 000 AIDS-related deaths [1]. The Joint United Nations Programme on HIV/AIDS (UNAIDS) developed an ambitious strategy to end AIDS as a global health threat by 2030, with specific goals of ensuring that 90% of people living with HIV/AIDS (PLWHA) are aware of their status, 90% of those who are aware of their status receive antiretroviral therapy, and 90% of those receiving antiretroviral therapy achieve viral suppression (90-90-90 targets) by 2020 [2] which has now been updated to 95-95-95 targets by 2025 [3]. In 2020, a global cascade demonstrated that 84% of people living with HIV knew their status, 73% of those living with HIV received therapy, and 66% were virally suppressed. In Thailand, 94% of people living with HIV were aware of their status, 84% received treatment, and 97% achieved viral suppression [4], falling short of the 90-90-90 targets.

HIV-related stigma and discrimination continue to endanger people living with the virus and keep millions of people from seeking testing, prevention, and treatment services [5,6], which are significant barriers to achieving global goals to end the HIV epidemic. Numerous studies have found that HIV-related stigma was associated with refusal to test for HIV, non-disclosure to partners, and low participation in biomedical prevention approaches [7-21]. Furthermore, stigma and discrimination affect people living with HIV in education systems, justice systems, workplaces, families and communities, and emergency and humanitarian settings [22]. Despite growing evidence on HIV stigma over the last two decades, both from research studies and programmatic experience [6,23-25], the stigma associated with infection remains. People living with HIV face enormous stigma and discrimination in society [26-34]. Poor HIV knowledge and awareness play a major role regarding the existence of stigma and discrimination associated with HIV/AIDS [34-36].

Thailand addressed this issue by adopting zero HIV stigma and discrimination as one of the major goals of Thailand National Operational Plan Accelerating Ending AIDS 2015-2019 [37] as well as establishing a goal for reducing HIV stigma and discrimination in the National AIDS Strategy 2017-2030 [38]. The action plan included a campaign to educate and raise public awareness about the issue, such as the zero-discrimination campaign promoted on television, radio, and social media platforms, as well as an intervention to reduce HIV-related stigma and discrimination in health care facilities. Standardized tools and methods for monitoring HIV stigma in health care facilities were also developed [39].

The National Health Examination Survey (NHES) is a Thai national demographic and health survey conducted roughly every four to five years. The previous survey, carried out in 2013-2014, showed that more than half of the general adult Thai population held stigmatizing attitudes toward PLWHA [28]. The focus of this study was a national survey on HIV stigma in the general population, which was part of the national survey. We describe and discuss relevant findings from the 6^th^ National Health Examination Survey regarding HIV stigma and discrimination among Thais and provide follow-up information on the country's progress in reducing HIV stigma and discrimination.

## METHODS

### Design and setting

The 6^th^ Thai National Health Examination Survey (NHES VI) was a large-scale survey carried out throughout the country during 2019-2020. The survey was conducted under the leadership of the National Health Examination Survey Office with the collaboration of academics from central and regional government universities. The survey aimed to determine the prevalence and risk factors of significant health conditions at the country level as well as the regional level. To identify potential participants, a four-staged probability sampling technique was used: 1) five provinces in each of the four regions of Thailand (north, central, northeast, and south), and Bangkok (mandatory site) were selected, 2) three to five districts were randomly selected from each province, 3) enumeration areas (EAs) in urban and rural areas were randomly selected from each province, for a total 540 EAs, and 4) individuals of both sexes from each age group (1-14 years, 15-59 years, and 60 years and over) were randomly selected from each EA. The final sample comprised 32 400 people of all ages from five regions, including Bangkok. Participants were divided into five age groups: 1-5 years, 6-9 years, 10-19 years, 20-59 years, and 60 years and older. This study was part of NHES VI, which was designed to survey HIV stigma in the general adult population aged 20 to 59.

### Measurements of HIV stigma

HIV stigma was assessed using six items, the same as in the previous round in 2013-2014 [28]. These included questions about HIV stigma domains [40]: 1) anticipated stigma (Question 1. Most people hesitate to take an HIV or AIDS test due to fear of people's reaction if the test result is positive for HIV.), 2) perceived stigma (Question 2. People living with or thought to be living with HIV or AIDS lose respect or social standing.), 3) fear of HIV infection (Question 3. Do you fear that you could contract HIV if you come into contact with the saliva of a person living with HIV?), 4) social judgment (Question 4. Do you agree with this sentence?: “I would be ashamed if someone in my family had HIV or AIDS”), 5) experienced stigma (Question 5. You feel too disgusted to buy fresh food or ready-to-eat food from a shopkeeper or vendor whom you know has HIV or AIDS.), and 6) discrimination (Question 6. You think that children living with HIV or AIDS should not attend the same classroom with other children.). All of these questions only accepted yes/no responses.

For questions 5 and 6, “yes” answers represented negative attitudes and “no” answers represented positive attitudes. The main study outcome was a composite indicator that combined the number of respondents who answered “yes” to question 5 and/or question 6. This composite indicator was recommended by UNAIDS, as the global indicator for discriminatory attitudes toward PLWHA in the general population [28,40].

Gender, age, religion, marital status, education, monthly income, urban/rural location (defined as those living outside of the municipality), and geographical region of the country in which they lived were all asked, as well as the HIV status of a respondent's relative or acquaintance and HIV testing history, which were new questions added to the questionnaire for this round of survey. The information was gathered through face-to-face interviews conducted by trained interviewers.

### Statistical methods

We weighted all analysis to consider the probability of sampling of the 2019 Thai population aged 20 to 59 years. We estimated proportions of stigmatizing attitudes for the entire population as well as subgroups based on sex, age, religion, marital status, education level, monthly income, urban/rural location, region, and the HIV status of a respondent's relative or acquaintance, as well as respondents' HIV testing history. The demographic characteristics of the participants were displayed as frequencies and percentages. We calculated the proportion of people who answered “yes” to each question, which was recognized as a stigmatizing attitude, and excluded the respondents who did not answer the questions from the denominators. The main study outcome was a composite indicator that combined the number of respondents who answered “yes” to question 5 and/or question 6 as the numerator. UNAIDS recommended this composite indicator as the global indicator for discriminatory attitudes toward PLWHA in the general population. We investigated the associations between demographic characteristics and the main outcome using χ^2^ tests with a 0.05 significance level. We also used univariable and multivariable logistic regression analyses to identify predictors of discriminatory attitude toward PLWHA based on the global indicator. We selected the variables with the significance level to be included in the multivariable model.

### Ethics approval

The study has been approved by the Committee on Human Rights Related to Research Involving Human Subjects at Mahidol University's Faculty of Medicine Ramathibodi Hospital, Thailand (Document No.COS.MURA2019/934). Prior to taking the surveys, all participants provided written informed consent.

## RESULTS

The survey included 11 843 adults aged 20 to 59, with 61.0% being female, the average age being 42 years old, 92.4% being Buddhists, 68.4% being married and living with their spouse, 48.3% having completed at least secondary school, 58.7% earning less than 10 000 baht (about US$280) per month, and 55.3% living in urban areas (**Table 1**). According to HIV status of a respondents' relative or acquaintance and their HIV testing history, 74.7% did not have an HIV/AIDS infected relative or acquaintance, 58.2% had never obtained an HIV test. For those who had previously received an HIV test, 66.2% had done so more than two years ago, and 50% had done so for reasons that were not specified, followed by a health check-up (36.7%) (**Table 1**).

**Table 1:** Characteristics of the respondents (n = 11 843)

Variables	n	Unweighted (%)	Weighted (%)
**Gender**			
Male	4620	39.0	48.9
Female	7223	61.0	51.1
**Age in years (mean = 42.08, SD = 11.538)**			
20-39	4939	41.7	41.2
40-49	2849	24.1	24.4
50-59	4055	34.2	34.4
**Religion**			
Buddhist	10 929	92.4	94.6
Christian	100	0.8	1.0
Islam	803	6.8	4.3
No religion	4	0.0	0.1
Not answered	7	-	-
**Marital status**			
Single	2586	21.8	21.3
Couple	8094	68.4	70.1
Widow/divorced/separated	1156	9.8	8.7
Not answered	7	-	-
**Education**			
None or primary only	4296	36.3	38.6
Secondary	5708	48.3	48.1
Certificate or higher	1828	15.4	13.3
Not answered	11	-	-
**Monthly income (Thai Baht)**			
<10 000	6955	58.7	58.6
≥10 000	4888	41.3	41.4
**Living area**			
Urban	6549	55.3	35.6
Rural	5294	44.7	64.4
**Region**			
North	2421	20.4	18.0
Central	2721	23.0	26.2
Northeast	2795	23.6	33.0
South	2549	21.5	14.1
Bangkok	1357	11.5	8.8
**Having an HIV/AIDS-infected relative or acquaintance**			
Yes	2834	23.9	23.8
No	8846	74.7	74.7
Not sure	163	1.4	1.5
**Had previously obtained an HIV test**			
Yes	4947	41.8	41.9
No	6896	58.2	58.1
If yes, last HIV testing (n = 4947)			
*Less than 1 y*	738	14.9	14.6
1-2 *y*	935	18.9	17.8
*More than 2 y*	3274	66.2	67.6
Reason for HIV testing (n = 4947)			
*Risky behavior*	279	5.6	6.2
*Checkup*	1815	36.7	37.8
*Apply for a job/health insurance*	380	7.7	8.9
*No reasons given*	2473	50.0	47.1

SD – standard deviation, HIV – human immunodeficiency virus, AIDS – acquired immunodeficiency syndrome

When ranking the proportion of negative attitude responses to all 6 questions from the highest to the lowest, 78.5% agreed that most people hesitate to take an HIV or AIDS test due to fear of people's reaction if the test result is positive for HIV, 66.6% thought that people living with or thought to be living with HIV or AIDS lose respect or social standing, 54.4% feared that they could contract HIV if they come into contact with the saliva of a person living with HIV, 41.0% felt too disgusted to buy fresh food or ready-to-eat food from a shopkeeper or vendor whom you know has HIV or AIDS, 28.2% agreed with the sentence “I would be ashamed if someone in my family had HIV or AIDS”, and 20.8% thought that children living with HIV or AIDS should not attend the same classroom with other children. Regarding the UNAIDS global indicator for discriminatory attitude, 48.6% responded negatively to either question 5 or question 6 (**Table 2**).

**Table 2:** Percentage and number of the respondents who had HIV stigmatizing attitudes toward PLWHA

Domain	Question	The 6^th^ NHES			the 5^th^ NHES [28]		
		**Answered “Yes”**		**Did not answer (n (%))**	**Answered “Yes”**		**Did not answer (n (%))**
		**Weighted %**	**n/N**		**Weighted %**	**n/N**	
Anticipated stigma	1. Most people hesitate to take an HIV or AIDS test due to fear of people's reaction if the test result is positive for HIV.	78.5	9340/11 841	2 (0.0)	76.9	8006/10 464	58 (0.6)
Perceived stigma	2. People living with or thought to be living with HIV or AIDS lose respect or social standing.	66.6	7773/11 841	2 (0.0)	69.2	7211/10 461	61 (0.6)
Fear of HIV infection	3. Do you fear that you could contract HIV if you come into contact with the saliva of a person living with HIV?	54.4	6477/11 841	2 (0.0)	57.0	6031/10 458	64 (0.6)
Social judgment	4. Do you agree with this sentence?: “I would be ashamed if someone in my family had HIV or AIDS”	28.2	3165/11 839	4 (0.0)	38.2	3931/10 459	63 (0.6)
Experienced stigma	5. You feel too disgusted to buy fresh food or ready-to-eat food from a shopkeeper or vendor whom you know has HIV or AIDS.	41.0	4900/11 841	2 (0.0)	52.1	5429/10 465	57 (0.5)
Discrimination	6. You think that children living with HIV or AIDS should not attend the same classroom with other children.	20.8	2415/11 840	3 (0.0)	23.7	2512/10 455	67 (0.6)
**Global indicator for discriminatory attitudes toward PLWHA (answered “yes” to either question 5 and/or question 6)**		48.6	5790/11 840	3 (0.0)	58.6	6108/10 451	71 (0.7)

NHES – National Health Examination Survey, HIV – human immunodeficiency virus, AIDS – acquired immunodeficiency syndrome, PLWHA – people living with HIV/AIDS

When examining the associations between demographic characteristics and the UNAIDS global indicator for discriminatory attitude toward PLWHA, we discovered significant differences between respondents' age groups (*P* < 0.001), religion (*P* < 0.001), marital status (*P* = 0.034), education (*P* = 0.005), and region (*P* < 0.001). However, discriminatory attitudes did not differ based on gender, monthly income, or living area (**Table 3**). Furthermore, when looking at the associations between HIV status of a respondents’ relative or acquaintance and HIV testing history for discriminatory attitude toward PLWHA, we found significant differences between the association of HIV status of a respondents' relative or acquaintance (*P* < 0.001), HIV testing history (*P* < 0.001), and reason for HIV testing (*P* = 0.012) (**Table 3**).

**Table 3:** Relationship between characteristics of the respondents and HIV stigmatizing attitudes toward PLWHA

Characteristics	Weighted % of negative attitudes												Global indicator for discriminatory attitudes toward PLWHA	
	**Anticipated stigma**		**Perceived stigma**		**Fear of HIV infection**		**Social judgment**		**Experienced stigma**		**Discrimination**			
	**%**	***P*-value**	**%**	***P*-value**	**%**	***P*-value**	**%**	***P*-value**	**%**	***P*-value**	**%**	***P*-value**	**%**	***P*-value**
**Gender**		0.639		0.009*		0.301		<0.001*		0.012*		0.006*		0.307
Male	78.7		67.9		53.7		30.8		39.4		22.8		47.8	
Female	78.3		65.4		55.1		25.8		42.7		19.0		49.4	
**Age (in years)**		0.077		0.187		<0.001*		<0.001*		<0.001*		<0.001*		<0.001*
20-39	78.9		67.6		57.6		24.9		43.1		20.0		50.1	
40-49	79.5		66.5		51.0		25.3		36.5		15.9		43.9	
50-59	77.2		65.5		53.1		34.2		41.8		25.2		50.3	
**Religion**		0.038*		0.031*		<0.001*		<0.001*		<0.001*		<0.001*		<0.001*
Buddhist	78.5		66.5		54.0		27.9		40.6		20.4		48.0	
Christian	68.7		57.6		48.8		21.1		40.5		19.9		53.0	
Islam	80.8		71.3		66.9		37.3		52.1		31.1		63.2	
**Marital status**		0.021*		0.488		0.649		<0.001*		0.002*		0.442		0.034*
Single	76.5		66.5		54.8		24.1		39.8		20.1		47.0	
Couple	79.1		66.9		54.5		29.8		42.1		21.1		49.5	
Widow/divorced/separated	78.4		65.4		53.0		25.3		35.7		20.1		45.9	
**Education**		<0.001*		0.187		0.056		<0.001*		0.068		<0.001*		0.005*
None/primary	75.8		66.5		54.1		32.9		41.1		26.0		50.2	
Secondary	79.7		67.4		55.5		26.4		41.8		17.9		48.4	
Certificate or higher	81.7		64.4		51.5		20.8		38.3		16.7		44.9	
**Monthly income (Thai Baht)**		0.031*		0.822		<0.001*		0.042*		0.102		0.631		0.260
<10 000	77.7		66.7		56.1		29.2		41.8		21.1		49.3	
≥10 000	79.6		66.5		52.1		26.7		40.0		20.4		47.8	
**Living area**		0.479		0.778		0.807		0.003*		0.450		0.111		0.298
Urban	79.0		66.4		54.2		25.6		40.3		19.8		47.7	
Rural	78.2		66.8		54.5		29.7		41.5		21.4		49.2	
**Region**		0.001*		<0.001*		<0.001*		<0.001*		<0.001*		0.104		<0.001*
North	78.3		57.8		45.1		21.4		36.3		18.9		44.1	
Central	78.5		67.4		51.3		24.6		35.1		19.4		43.3	
Northeast	78.7		71.5		59.5		35.0		46.9		21.5		53.0	
South	81.7		63.0		59.6		26.7		41.8		24.5		51.5	
Bangkok	73.3		69.8		55.5		29.6		45.5		20.8		53.1	
**Having an HIV/AIDS-infected relative or acquaintance**		0.142		<0.001*		<0.001*		<0.001*		<0.001*		0.011*		<0.001*
Yes	79.1		58.4		45.0		19.4		30.2		17.3		39.1	
No	78.5		69.4		57.5		30.9		44.7		22.0		51.9	
Not sure	71.2		59.4		51.2		33.7		33.4		16.9		38.8	
**Had previously obtained an HIV test**		<0.001*		0.489		<0.001*		<0.001*		0.001*		0.005*		<0.001*
Yes	80.6		66.2		51.7		26.5		39.0		18.8		46.4	
No	77.0		66.9		56.4		29.4		42.5		22.3		50.3	
**Reason for HIV testing**		0.060		<0.001*		0.020*		0.028*		0.049*		0.103		0.012*
Risky behavior	85.2		76.2		58.4		34.8		39.5		18.6		45.0	
Checkup	79.0		63.7		47.9		25.8		36.8		18.9		43.9	
Apply for a job or health insurance	79.0		75.5		52.5		29.3		35.9		13.2		41.2	
No reasons given	81.6		65.2		53.6		25.4		41.3		19.9		49.6	

HIV – human immunodeficiency virus, AIDS – acquired immunodeficiency syndrome, PLWHA – people living with HIV/AIDS

*Statistically significant.

Univariable and multivariable analyses of factors associated with the UNAIDS global indicator on discriminatory attitudes toward PLWHA showed similar results. Independent predictors of discriminatory attitudes included being aged 20-39 (adjusted odds ratio (aOR) = 1.32, 95% confidence interval (CI) = 1.18-1.47) or 50-59 (aOR = 1.23, 95% CI = 1.09-1.40) as compared to being aged 40-49, being Muslim compared to being Buddhist (aOR = 1.73, 95% CI = 1.46-2.06), being married compare to being single (aOR = 1.15, 95% CI = 1.04-1.28), holding a certificate degree or higher compared to not studying or being enrolled in primary education (aOR = 0.81, 95% CI = 0.68-0.97), living in the Northeast (aOR = 1.27, 95% CI = 1.12-1.45) and Bangkok (aOR = 1.30, 95% CI = 1.12-1.51) compared to living in the North. Furthermore, having no HIV/AIDS infected relative or acquaintance compared to having an HIV/AIDS infected relative or acquaintance (aOR = 1.56, 95% CI = 1.41-1.73) and not obtaining an HIV test (aOR = 1.10, 95% CI = 1.02-1.19) compared to those who did (**Table 4**).

**Table 4:** Univariable and multivariable analysis of factors associated with the UNAIDS global indicator on discriminatory attitudes toward PLWHA

Independent variables	Crude OR	*P*-value (95% CI)	Adjusted OR	*P*-value (95% CI)
**Gender**				
Male	1.00			N/A
Female	1.07	0.307 (0.94-1.21)		
**Age (years)**				
20-39	1.28	<0.001 (1.17-1.41)*	1.32	<0.001 (1.18-1.47)*
40-49	1.00		1.00	
50-59	1.29	<0.001 (1.15-1.46)*	1.23	0.003 (1.09-1.40)*
**Religion**				
Buddhist	1.00		1.00	
Christian	1.22	0.305 (0.82-1.83)	1.14	0.497 (0.76-1.73)
Islam	1.86	<0.001 (1.60-2.17)*	1.73	<0.001 (1.46-2.06)*
**Marital status**				
Single	1.00		1.00	
Couple	1.10	0.042 (1.00-1.22)*	1.15	0.010 (1.04-1.28)*
Widow/divorced/separated	0.96	0.551 (0.83-1.11)	1.02	0.834 (0.86-1.20)
**Education**				
None or primary	1.00		1.00	
Secondary	0.93	0.119 (0.85-1.02)	0.91	0.060 (0.82-1.01)
Certificate or higher	0.81	0.003 (0.71-0.92)*	0.81	0.023 (0.68-0.97)*
**Monthly income (Thai Baht)**				
<10 000	1.00			N/A
≥10 000	0.94	0.026 (0.85-1.05)		
**Living area**				
Urban	1.00			N/A
Rural	1.06	0.298 (0.94-1.19)		
**Region**				
North	1.00		1.00	
Central	0.97	0.663 (0.82-1.13)	0.91	0.143 (0.79-1.04)
Northeast	1.43	<0.001 (1.22-1.66)*	1.27	0.001 (1.12-1.45)*
South	1.34	0.007 (1.10-1.65)*	1.07	0.411 (0.90-1.27)
Bangkok	1.43	<0.001 (1.23-1.67)*	1.30	0.002 (1.12-1.51)*
**Having an HIV/AIDS infected relative or acquaintance**				
Yes	1.00		1.00	
No	1.68	<0.001 (1.50-1.88)*	1.56	<0.001 (1.41-1.73)*
Not sure	0.99	0.904 (0.70-1.39)	0.97	0.889 (0.96-1.37)
**Had previously obtained an HIV test**				
Yes	1.00		1.00	
No	1.17	<0.001 (1.08-1.25)*	1.10	0.016 (1.02-1.19)*

OR – odds ratio, CI – confidence interval, HIV – human immunodeficiency virus, AIDS – acquired immunodeficiency syndrome

*Statistically significant.

## DISCUSSION

This study was a national survey on HIV stigma and discrimination in the general population that was conducted as part of the 6^th^ NHES, which used rigorous methodology and quality control. Because the study was a population-based probability sampling survey and since all the figures were weighted, the results should accurately represent the characteristics of the general Thai adult population. We found that 48.6% of Thai adults had discriminatory attitudes toward PLWHA based on the UNAIDS definition. Despite Thailand seemingly having a lower rate of discrimination than according to UNAIDS 2020 data from 25 of 36 countries (showing that more than 50% of people aged 15-49 have discriminatory attitudes toward PLWHA) [41], HIV-related stigma and discrimination in the country's general population may continue to pose challenges to HIV/AIDS ending efforts.

Regarding the question that comprised the global discrimination composite indicator, 41.0% of Thai adults agreed that they were too disgusted to buy fresh or ready-to-eat food from an HIV-positive vendor. This figure was lower than the 53.5% reported in a survey of young female migrant workers in Vietnam [33] and the 50.0% reported in a Nigerian population survey [34]. However, it was higher than the 12.1% reported in Botswana [35]. One-fifth of Thai adults believed that children with HIV or AIDS should not be placed in the same classrooms as other children. Even though it was the smallest proportion of all six questions, its significance is high because it reflects a discriminatory attitude toward vulnerable populations [28] and may have an impact on children's enrolment and attendance in school [42,43] and their educational outcomes [44]. It is critical that educators, parents, and students collaborate to protect HIV/AIDS-affected children's right to an education. Public health educational campaigns emphasizing non-transmission knowledge and furthering family education in conjunction with school education may help to establish a non-discriminatory environment and safeguard public support programs for educational rights in the future [43].

Concerning other HIV stigma domain-related questions, nearly 80.0% of Thai adults agreed that most people are hesitant to take an HIV test because they are afraid of how others will react if the test results are positive, which is the highest proportion of all questions. This high level of anticipated stigma is concerning because it may prevent people from getting HIV tests, even if they are at high-risk. Furthermore, it would be a significant barrier to HIV testing for those at risk for HIV, potentially contributing to the failure of the goal of ending AIDS, as 95% of people living with HIV/AIDS are unaware of their status.

Perceived stigma refers to how much a member of society expects PLWHA to face prejudice and discrimination from other community members. This stigma domain had the second highest proportion (66.6%) in our study. Concerning the fear of HIV infection, the findings revealed that 54.4% of Thai adults still had misconceptions about HIV transmission mode, leading to fear of acquiring HIV through casual contact with PLWHA. Similar to the findings of the Indonesian general population survey, 75.9% of respondents had misconceptions about HIV transmission [45]. Misconceptions about how HIV spreads also contribute to stigma and discrimination against PLWHA. This finding suggests that there is an urgent need to increase efforts on social and behavioral change communication, as well as to improve access to quality comprehensive HIV transmission and prevention education. Furthermore, HIV/AIDS messaging must be of higher quality and more comprehensive. Regarding social judgment, 28.2% of Thai adults agreed that they would be embarrassed if someone in their family had HIV or AIDS. Despite its small size, it could be one of the potential barriers to HIV testing, treatment, and disclosure of HIV positive status.

Compared to the previous national survey [28], discriminatory attitudes toward PLWHA among Thai adults appear to have declined, falling from 58.6% to 48.6%. When examining each question according to the HIV stigma domains, most of those indicating negative attitudes toward PLWHA have also decreased. Perceived stigma fell from 69.2% to 66.6%, while fear of HIV infection fell from 57.0% to 54.4% and social judgment fell from 38.2% to 28.2%. Concerning the global discrimination composite indicator, both experienced stigma and discrimination appear to have decreased compared to the previous survey (from 52.1% to 41.0 and 23.7% to 20.8%, respectively). These findings may serve as preliminary confirmation that Thailand is on track for reducing HIV stigma and discrimination. However, the results revealed that one HIV stigma domain appears to have decreased. Anticipated stigma increased from 76.9% in the previous round to 78.5% this round. This finding is troubling and should be addressed because HIV testing is an important entry point into HIV prevention, care, and treatment, as well as a critical component of AIDS-eradication efforts. Efforts from all over the world to end AIDS will be futile if most people continue to fear stigma from others if their HIV test results are positive.

According to multivariable analysis, we found that people aged 40-49 had lower discriminatory attitude toward PLWHA compared to younger (20-39) and older (50-59) age groups. This could be explained, as it was in the previous survey round [28], by the fact that people in this age group entered adolescence and young adulthood during Thailand's HIV epidemic in the early 1990s. Direct experience with the loss of loved ones to AIDS, and exposure to extensive education at the time may have fostered greater sympathy toward PLWHA, explaining the group's less discriminatory attitude. We also found that HIV stigma and discrimination were more prevalent in those who self-identified as Muslim. This could be due to religious beliefs regarding unacceptable sex acts and/or drug-related practices [46-48]. This finding also suggested that more research and interventions to identify the actual causes and means to alleviate HIV and HIV stigma among the country's Muslim communities should be carried out.

We also discovered that married Thai adults had more discriminatory attitude than those who had never married. This is similar to studies conducted in Ethiopia [49] and Nigeria [34], but contradicts studies conducted in sub-Saharan Africa [50], which found that married individuals have a lower discriminatory attitude toward PLWHA than singles. It is possible that those who had married had a more discriminatory attitude toward PLWHA because most married people are older and may have faced HIV/AIDS stigma through their experiences. Thai adults who had never studied or had only a primary level of education were more discriminative than those with a certificate or higher education, which is consistent with previous research [34,49,51,52]. Individuals who are uneducated or have a low level of education may not understand the disease, including misconceptions about modes of transmission. It may lead to negative attitudes toward HIV-infected people, potentially increasing HIV-related stigma and discriminatory attitudes toward PLWHA. This study also revealed that discriminatory attitude was prevalent in Bangkok and the Northeast, which was consistent with the previous round of the national survey. It should be noted, however, that living in the south, where the majority of Thai Muslims reside, was not associated with discriminatory attitudes toward PLWHA in the multivariable model. This finding demonstrated that the predictor variables had an independent effect on the outcome and should be targeted separately [28].

Findings highlighted that Thai adults who did not have an HIV/AIDS infected relative or acquaintance have a more discriminatory attitude toward PLWHA than those who did. It is possible that having an HIV/AIDS infected relative or acquaintance resulted in a less discriminatory attitude toward PLWHA because they gained an understanding of HIV-infected people as well as sympathy for PLWHA through living, communicating, and discussing with their relative or acquaintance. Furthermore, Thai adults who had previously obtained an HIV test experienced less HIV discrimination than those who had not. People who get HIV tests for any reason must go through an HIV counselling process with health care professionals who will teach them about the disease and advise them on what to do if their test results are positive. HIV counselling improves people's understanding of the disease, which leads to a less discriminatory attitude toward HIV-infected people. This could be conclusive evidence that HIV counselling in the country is effective in terms of clients learning more about the disease during the process.

Although HIV stigmatizing and discriminatory attitudes toward PLWHA appear to be decreasing among Thai adults, there are still concerns. More than three decades after the country's HIV epidemic began, Thailand has succeeded in reducing new infections, particularly in the last decade [53-55], but stigmatizing attitudes toward PLWH persist among the general population. Thailand has addressed this issue and set the goal of reducing HIV stigma and discrimination in the National AIDS Strategy since 2012. The Ministry of Public Health supports and implements many activities to reduce HIV-related stigma and discrimination, including a campaign to educate the public and raise awareness about the issue and an intervention to reduce HIV-related stigma and discrimination in health care facilities. Increasing public awareness of HIV prevention and treatment through those campaigns may help reduce fear of casual contact. However, a previous study suggested that educational efforts alone may not be effective in changing attitudes such as fear and that a multifaceted approach may be needed [56]. Further qualitative research should be conducted to gain a better understanding of the most effective approaches for reducing stigmatizing and discriminatory attitudes toward PLWHA in the country, particularly among young adults, the elderly, Thai Muslims, married people, and the uneducated, who were found to have a high prevalence of HIV discriminatory attitudes in this study. These and other efforts to monitor and reduce stigmatizing attitudes toward PLWHA are required to ensure continued progress in increasing HIV prevention, testing, and treatment.

### Limitations

This study is the second national survey involving HIV stigma and discrimination, and it is part of the 6^th^ Thai National Health Examination Survey. All figures were weighted to reflect the actual population, so the results should accurately represent Thai adult population characteristics. However, our study has some limitations. First, there is limited comparability with other surveys using global indicators because some questions were adapted and the respondent age range (20-59) did not correspond to the age range for global recommendations (15-49), as well as limited comparability with other surveys using a variety of questions and methodologies. Second, the study had to rely on NHES data, which was intended to capture only a broad picture of Thai population health. This is why other significant variables related to HIV-related stigma and discrimination, such as HIV knowledge, sexual orientation, and key population status of respondents, and their risk behaviors, were not measured in this study. Finally, as with other surveys focusing on sensitive social issues, social desirability bias may be an issue, leading to under-reporting of discriminatory attitudes.

## CONCLUSIONS

We found that HIV stigmatizing and discriminatory attitudes toward PLWHA among Thai adults were decreasing but remained concerning. A campaign to educate and raise public awareness about the issue and an intervention to reduce HIV-related stigma and discrimination in health care facilities in the country must be maintained. Further research would help understand which approaches are the most effective at reducing stigmatizing and discriminatory attitudes toward PLWHA in the country, particularly among young adults, the elderly, Thai Muslims, married people, and the uneducated, who were found to have a high prevalence of HIV discriminatory attitudes in this study. These and other efforts to monitor and reduce stigmatizing attitudes toward PLWHA are required to ensure continued progress in increasing HIV prevention, testing, and treatment.
